# Generative adversarial networks synthetic optical coherence tomography images as an education tool for image diagnosis of macular diseases: a randomized trial

**DOI:** 10.3389/fmed.2024.1424749

**Published:** 2024-07-10

**Authors:** Jie Peng, Xiaoling Xie, Zupeng Lu, Yu Xu, Meng Xie, Li Luo, Haodong Xiao, Hongfei Ye, Li Chen, Jianlong Yang, Mingzhi Zhang, Peiquan Zhao, Ce Zheng

**Affiliations:** ^1^Department of Ophthalmology, Xinhua Hospital Affiliated to Shanghai Jiao Tong University School of Medicine, Shanghai, China; ^2^Joint Shantou International Eye Center of Shantou University and the Chinese University of Hong Kong, Shantou University Medical College, Shantou, China; ^3^Department of Ophthalmology, Shanghai Children’s Hospital, School of Medicine, Shanghai Jiao Tong University, Shanghai, China; ^4^School of Biomedical Engineering, Shanghai Jiao Tong University, Shanghai, China; ^5^Institute of Hospital Development Strategy, China Hospital Development Institute Shanghai Jiao Tong University, Shanghai, China

**Keywords:** medical education, macular diseases, generative adversarial networks, optical coherence tomography, resident training

## Abstract

**Purpose:**

This study aimed to evaluate the effectiveness of generative adversarial networks (GANs) in creating synthetic OCT images as an educational tool for teaching image diagnosis of macular diseases to medical students and ophthalmic residents.

**Methods:**

In this randomized trial, 20 fifth-year medical students and 20 ophthalmic residents were enrolled and randomly assigned (1:1 allocation) into Group real OCT and Group GANs OCT. All participants had a pretest to assess their educational background, followed by a 30-min smartphone-based education program using GANs or real OCT images for macular disease recognition training. Two additional tests were scheduled: one 5 min after the training to assess short-term performance, and another 1 week later to assess long-term performance. Scores and time consumption were recorded and compared. After all the tests, participants completed an anonymous subjective questionnaire.

**Results:**

Group GANs OCT scores increased from 80.0 (46.0 to 85.5) to 92.0 (81.0 to 95.5) 5 min after training (*p* < 0.001) and 92.30 ± 5.36 1 week after training (*p* < 0.001). Similarly, Group real OCT scores increased from 66.00 ± 19.52 to 92.90 ± 5.71 (*p* < 0.001), respectively. When compared between two groups, no statistically significant difference was found in test scores, score improvements, or time consumption. After training, medical students had a significantly higher score improvement than residents (*p* < 0.001).

**Conclusion:**

The education tool using synthetic OCT images had a similar educational ability compared to that using real OCT images, which improved the interpretation ability of ophthalmic residents and medical students in both short-term and long-term performances. The smartphone-based educational tool could be widely promoted for educational applications.

**Clinical trial registration**: https://www.chictr.org.cn, Chinese Clinical Trial Registry [No. ChiCTR 2100053195].

## Introduction

The application of medical knowledge is one of the crucial parts defined by the Accreditation Council for Graduate Medical Education (ACGME) for evaluating competency as an educational outcome of residency programs (1). For example, the timely and precise diagnosis of different macular diseases was vital to patients’ early treatment and vision preservation. Therefore, the diagnosis of macular diseases is an important part of residency training in China^1^ and other countries (2).

Among various macular diseases, the leading causes of blindness worldwide are age-related macular degeneration (AMD) and diabetic macular edema (DME). AMD accounts for 6–9% of legal blindness in industrialized countries (3). AMD is characterized by drusen along with progressive degeneration of photoreceptors and adjacent tissues (4). At any stage of AMD, new vessels may invade the outer retina and beyond, resulting in macular choroidal neovascularization (CNV), which is the hallmark lesion of neovascular AMD (5, 6). In addition, 1 out of 15 patients with diabetes suffers from DME (7), which is a vision-threatening form of diabetic retinopathy (DR). DME is characterized by exudative fluid accumulation in the macula (7). Optical coherence tomography (OCT) is a non-invasive technique for cross-sectional tissue imaging, providing real-time imaging and quantitative information *in vivo* (8), which is a standard diagnostic tool for many macular diseases (9). Therefore, ophthalmology residents must learn how to interpret an OCT scan and provide an accurate diagnosis report.

Education is the future. Education in medical image interpretation is an ongoing need that is addressed by text-based teaching and hands-on training (10). These traditional methods of education rely on large, diverse, and real medical images, which may be limited in medical schools or training institutions in remote areas or developing countries (11). Artificial intelligence (AI), especially deep learning (DL) algorithms, provides new tools to solve medical problems, such as imaging detection and disease diagnosis in AMD and DR (12–15). More recently, generative DL methods, such as generative adversarial networks (GANs), have shown their capability to generate realistic medical imaging, such as retinal fundus (16), computed tomography (17), and skin lesions (18).

Since biometric data, including retinal images, are personally identifiable information, they may be protected from inappropriate access regardless of the participant’s consent or local institutional review board (IRB) permission (19). The privacy of patients is a critical issue during the whole medical process. To avoid potential violations of privacy regulations, we previously proposed GANs architectures capable of synthesizing realistic OCT images. These images satisfactorily serve as training datasets for DL algorithms and education tools for retinal specialists and students (20). The use of GAN has benefited various tasks in the ophthalmology image domain (21). However, the adoption of GAN in ophthalmology is still in a very early stage of clinical validation, and GANs’ potential to aid medical education has not been explored. Therefore, this study aimed to test its utility as an educational tool in the real world as well as compare GANs OCT images to real OCT images.

## Materials and methods

### Ethical approval

With approval from the Institutional Review Board at Xinhua Hospital Affiliated with Shanghai Jiao Tong University (XHEC-D-2022-067), this trial was registered in the Chinese Clinical Trial Registry (No.ChiCTR 2100053195). Written informed consent was obtained from each participant, and the study adhered to the tenets of the Declaration of Helsinki.

### Sample size

The estimated sample size was calculated to detect a 10% (approximately 8 scores) improvement after training with GANs OCT, with 80% power and α = 0.05, assuming a precision of 8 SD. It was estimated that 16 participants were required in each group. Assuming a dropout rate of 20%, 20 participants were enrolled in two groups.

### Subjects and randomization

In this randomized trial, 20 fifth-year medical students from the Shenzhen University School of Medicine and 20 residents who participated in ophthalmic resident training at the ophthalmology department, Xinhua Hospital Affiliated with Shanghai Jiao Tong University School of Medicine in Shanghai, were enrolled into two groups (Group real OCT and Group GANs OCT) in March 2022. Xinhua Hospital is a tertiary medical center, which can provide retinal subspecialty care.

The participants were further randomly assigned (1:1 allocation) to two subgroups (Group real OCT and Group GANs OCT) using a random number table. In Group real OCT, residents and students learned about retinal diseases through an e-learning platform using real OCT images. In Group GANs OCT, participants learned retinal diseases using GANs synthetic OCT images with the same platform.

### The e-learning platform using real and GANs synthetic OCT images

**Figure 1:**
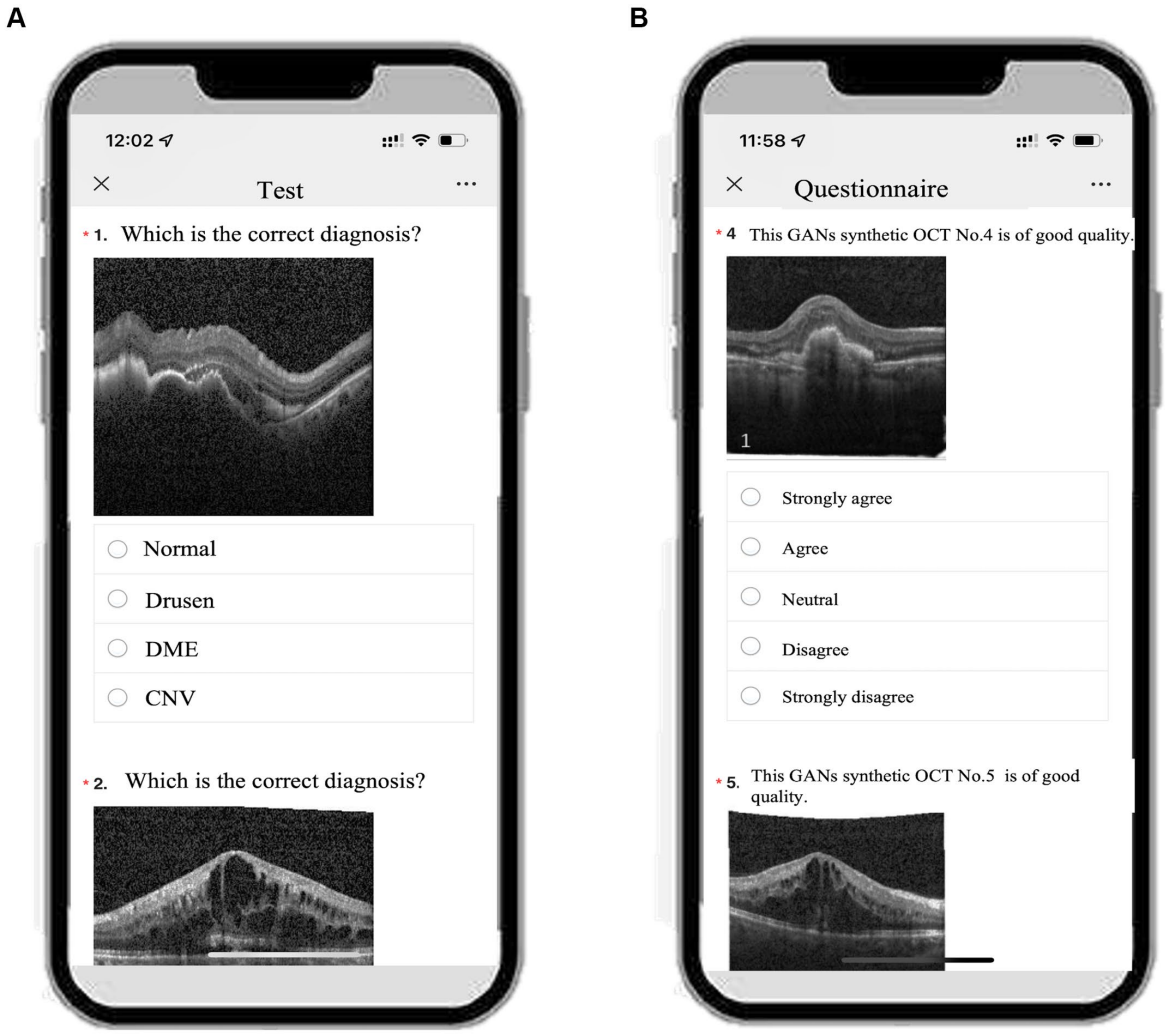
An example of the smartphone-based educational tool with either GANs or real OCT image with single choice questions and the questionnaire. **(A)** Screenshot of test1/2/3 on a smartphone. **(B)** Screenshot of the questionnaire on the same smartphone.

In the current study, we adopted Wenjuanxing^2^ to build the e-learning platform. Wenjuanxing is an online crowdsourcing platform in China that provides functions equivalent to those of Amazon Mechanical Turk (22). Using Wenjuanxing, we programmatically direct OCT images with retinal diseases as single-choice questions (SCQs), which students and residents can complete as smartphone-based e-learning resources (Figure 1).

OCT imaging is currently a standard of care for guiding the diagnosis and treatment of macular diseases (9). This technique uses light to capture high-resolution *in vivo* optical cross-sections of the retina. We collected OCT images of two leading blinding macular diseases worldwide: AMD and DME. The whole e-learning OCT images were downloaded from an opening dataset (23), including 37,206 OCT images from eyes with CNV, 11,349 OCT images from eyes with DME, 8,617 OCT images from eyes with drusen, and 51,140 OCT images from normal eyes.

**Figure 2:**
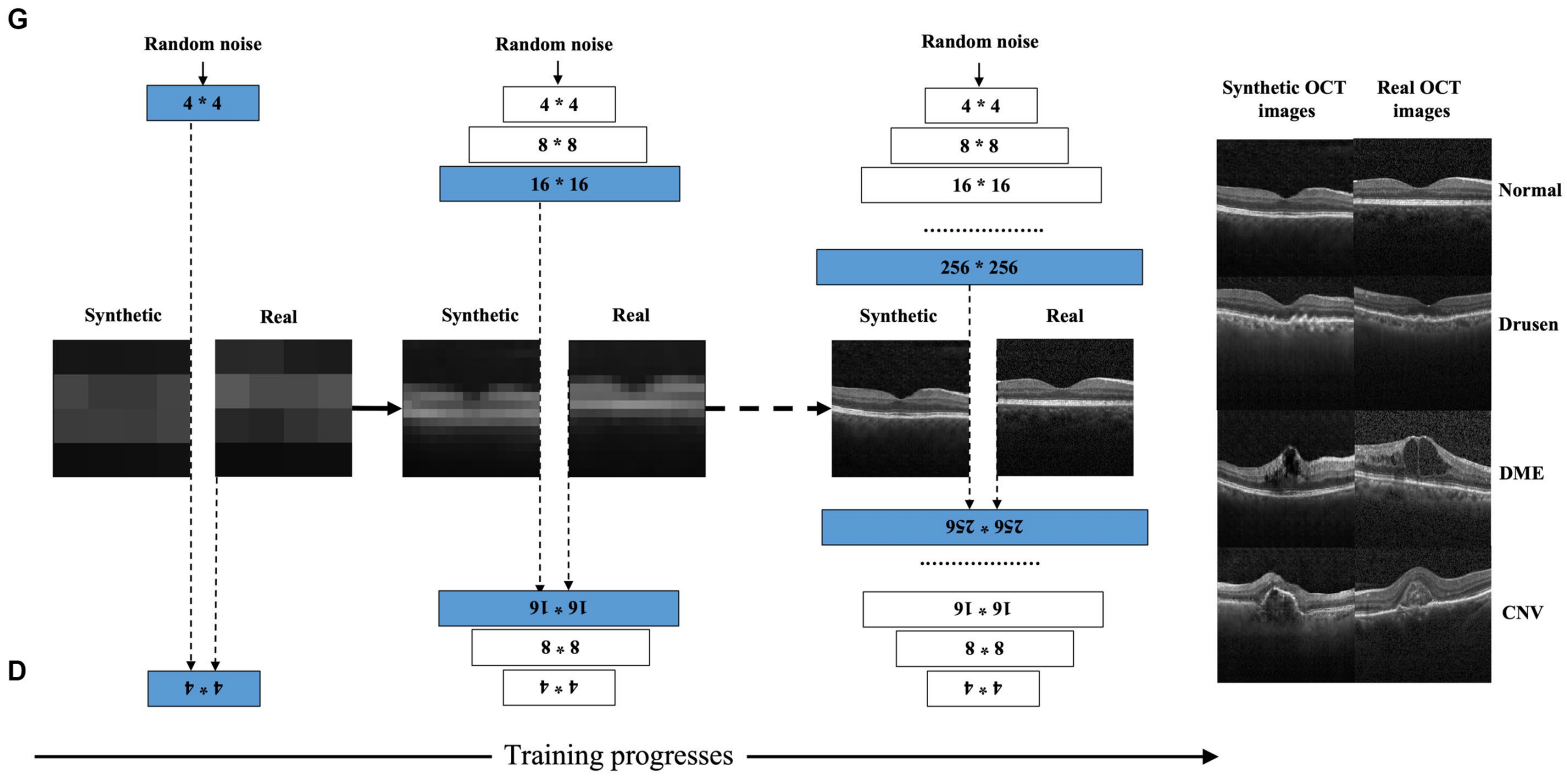
Schematic for generating synthetic OCT images by progressively growing generative adversarial networks. PGGANs starts at low resolution (4 × 4-pixel images), doubling to 8 × 8, 16 × 16, and so on until the desired output resolution (256 × 256 in the current study) is reached.

We adopted progressively growing generative adversarial networks (PGGANs) to synthesize high-resolution OCT images. We had reported the detailed description of the PGGANs in our previous study (20). In brief, PGGANs are an extension of the GANs architecture which is adversarially trained to perform antagonistic tasks, including a discriminative network (D) to discriminate between real and synthetic images and a generative network (G) to generate synthetic images ideally realistic enough to fool network D. During GANs training, the PGGANs start at low resolution (4 × 4-pixel images), doubling to 8 × 8, 16 × 16, and so on until the desired output resolution (256 × 256 in the current study) is reached. A schema of the PGGANs architecture can be seen in Figure 2. Our previous study showed good equivalents between real and synthetic images using the Frechet inception distance (FID) score (FID was 5.49 and 10.70 for real and synthetic OCT images, respectively) (20). We followed the scheme as described by our previous study. With each phase, an upsampling layer and a pair of convolutional layers were added to both the D and G networks. An upsampling layer involves two convolutional layers with 3 × 3 and 4 × 4 filters and a Leaky ReLU activation function (slope = 0.2). An average pooling is used for downsampling. We implement the PGGANs model with the TensorFlow framework (Google, version 2.1.0) (24) and Keras API (version 2.2.4). The computer platform was equipped with an NVIDIA (Santa Clara, CA) GTX 1080 Ti 12-GB GPU with an Intel (Santa Clara, CA) Core i7-2700K processor, 4.6-GHz CPU, and 128 GB RAM.

All images used were diagnosed and checked by three retina specialists.

### Study protocol

We first evaluate the subjects’ performance to identify macular diseases with a pretest (test 1), including 50 SCQs using real OCT images. The students were asked to choose the correct answer for OCT images, whether they thought the macular disorders were present or not. Following the OCT image analysis pretest, subjects of two subgroups received a 30-min smartphone-based education program for macular disease recognition training. The education program involved an OCT image atlas of various macular disorders. In the subgroup real OCT, residents and students learned macular diseases using real OCT images, whereas participants in Group GANs OCT learned with the same education program using GANs synthetic OCT images only. These included 12 OCT images from eyes with CNV, 12 OCT images from eyes with DME, 12 OCT images from eyes with drusen, and 12 OCT images from normal eyes.

**Figure 3:**
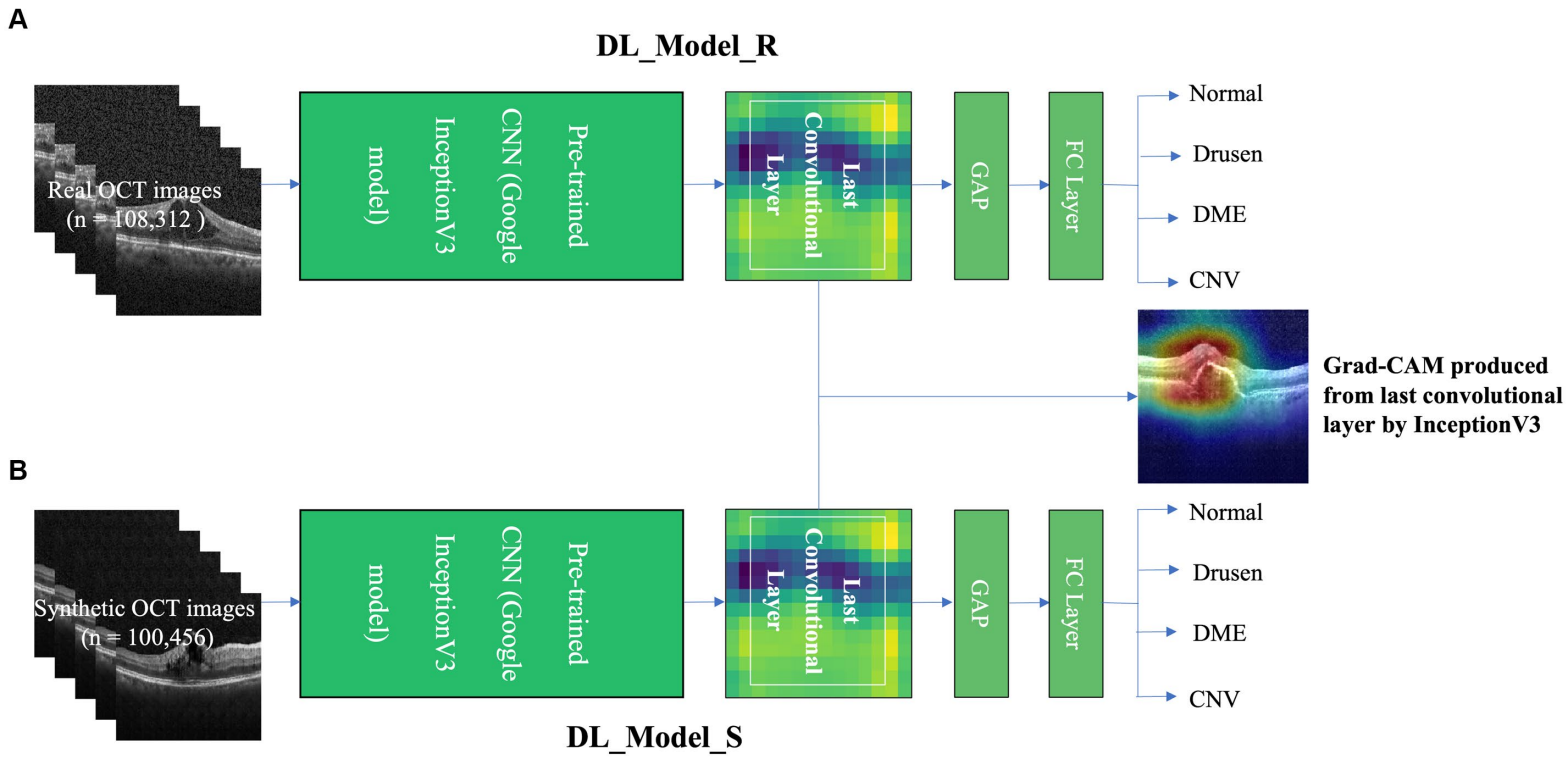
The grad-CAM and deep CNNs architecture. Two deep CNNs were trained on either real training with real(R) OCT images (DL_Model_R) **(A)** or training with synthetic(S) OCT images datasets (DL_Model_S) **(B)**. Grad-CAM was produced from the last convolutional layer by the InceptionV3 model.

To help trainees interpret the macular pathology in OCT images, we further apply the gradient-weighted class activation map (Grad-CAM) concept to highlight essential regions in different retinal disorders. Grad-CAM is a technique to provide a visual explanation of the network model. Our previous study demonstrated that Grad-CAM could facilitate clinical translation if the learning process is pathology-driven, not imaging device-driven (25). Briefly, we used either real (*n* = 108,312) or synthetic (*n* = 100,456) OCT datasets to train two deep convolutional neural networks (CNNs), and the area under the curves (AUCs) ranged from 0.90 to 0.99 (20), indicating excellent diagnostic performance. Grad-CAM was further produced from the class-specific gradient information that flows into the final convolutional layer of CNNs. The proposed grad-CAM algorithm is schematically depicted in Figure 3.

**Figure 4:**
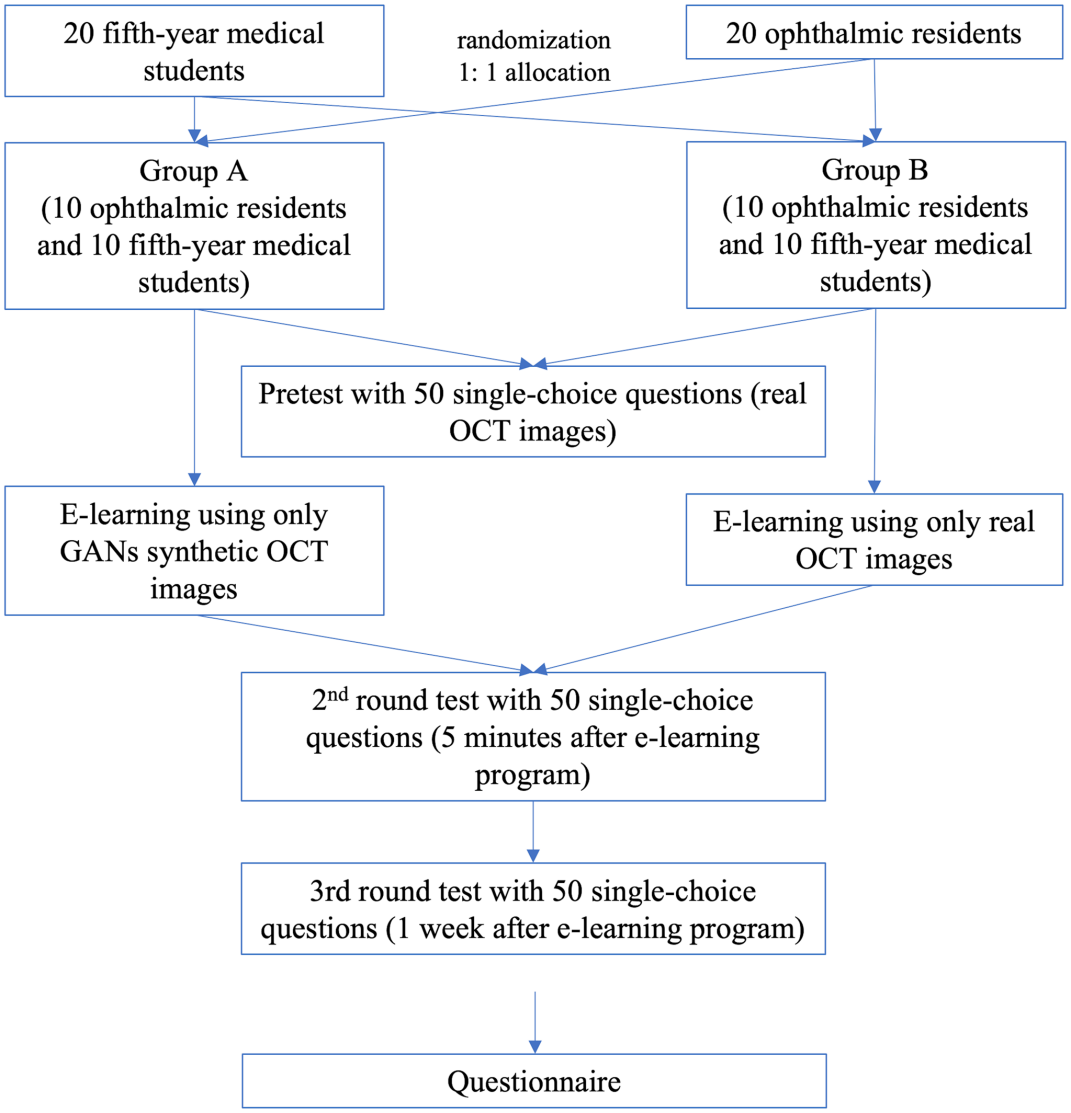
The chronological flowchart for the three tests and the learning process in the two groups.

To evaluate the short-term and long-term effects of our e-learning program, we further arranged two rounds of testing, which included two different sets of 50 SCQs. The second round of testing (test 2) was conducted 5 min after the education program. Each subgroup underwent another post-lecture test (the third round test, test 3) 1 week after the education program and the second one. The flowchart of the three tests and the learning process of the two groups are specified in Figure 4. After the final test, questionnaires were collected. Score improvement is defined as score gains compared to baseline (test 1). For better understanding and comparisons, the participants were subdivided into four subgroups as follows: Group 1: resident + GANs OCT; Group 2: resident + real OCT; Group 3: medical student+ GANs OCT; and Group 4: medical student + real OCT. In addition, Groups 1 and 3 are included in Group GANs OCT, while Groups 2 and 4 are in Group real OCT, Groups 1 and 2 are in Group residents, and Groups 3 and 4 are in Group medical students, respectively. Test time reduction is defined as time reductions compared to baseline (test 1). We evaluate the teaching effect of the tools in terms of the increased scores and test time reduction before and after e-learning. Assessment of the educational tool by participants was acquired in the questionnaires (Table 1).

**Table 1:** Twenty-one item questionnaire.

No.	Question
One-choice questions (5 = strongly agree/very good; 4 = agree/good; 3 = neutral/so-so; 2 = disagree/bad; 1 = strongly disagree/very bad)	
1	This GANs synthetic OCT No.1 is of good quality.
2	This GANs synthetic OCT No.2 is of good quality.
3	This GANs synthetic OCT No.3 is of good quality.
4	This GANs synthetic OCT No.4 is of good quality.
5	This GANs synthetic OCT No.5 is of good quality.
6	This GANs synthetic OCT No.6 is of good quality.
7	This GANs synthetic OCT No.7 is of good quality.
8	This GANs synthetic OCT images were hard to tell from the real ones.
9	This GAN/Real OCT image-based educational tool helps me to learn more OCT-associated knowledge.
10	This GAN/Real OCT image-based educational tool helps me to learn typical features of macular diseases in OCT images.
11	This GAN/Real OCT image-based educational tool can focus on the key point of OCT education.
12	This GAN/Real OCT image-based educational tool is more efficient than traditional lessons.
13	This GAN/Real OCT image-based educational tool helped to motivate the students to autonomous learning.
14	This GAN/Real OCT image-based educational tool helped to improve the students’ learning ability.
15	This GAN/Real OCT image-based educational tool helped to improve the students’ imaging diagnosis ability in a short time with limited images.
16	After the training, I am more confident in OCT imaging diagnosis of macular diseases.
17	This GAN/Real OCT image-based educational tool is well-designed.
18	This GAN/Real OCT image-based educational tool is quite satisfactory.
19	This GAN/Real OCT image-based educational tool should be promoted in other aspects of education.
20	I will recommend this GAN/Real OCT image-based educational tool to my peers.
Open-ended question	
21	Comparing to traditional speech, what are the advantages and disadvantages of these GAN/Real OCT image-based educational tool?

GAN, generative adversarial networks; No., number; OCT, optical coherence tomography.

### Statistics

Statistical analysis was performed using Statistical Package for the Social Sciences (SPSS) Version 26 (IBM Corp, Armonk, NY, United States). The data were expressed as the mean ± standard deviation (SD) or as median and range. The Shapiro–Wilk test was performed to examine whether the variables were distributed normally. Student’s t-test or the Wilcoxon Mann–Whitney test was used for comparisons between the subgroups according to data distribution. A paired *t*-test was applied to compare the scores between different rounds. A *p*-value was defined as statistically significant if *p* < 0.05.

## Results

### Results of the study

A total of 20 residents and 20 fifth-grade medical students were enrolled and finished the whole study. Test time duration of the three tests, scores of the three tests, score improvements and test time reduction, and comparisons between different groups are listed in Table 2.

**Table 2:** Results of the study.

							Group GAN OCT	Group real OCT		Group residents	Group medical students	
Items	Group 1	Group 2	*p*	Group 3	Group 4	*p*	Group 1 + 3	Group 2 + 4	*p*	Group 1 + 2	Group 3 + 4	*p*
Test time of test 1	628.70 ± 453.19	552.80 ± 327.50	0.673^a^	286.00 ± 163.30	286.10 ± 130.59	0.673^b^	409.00 (216.25, 673.75)	323.00 (238.25, 587.50)	0.989^b^	446.00 (314.75, 711.25)	286.05 ± 143.91	0.001^b^
Scores of test 1	84.20 ± 9.50	79.40 ± 14.42	0.391^a^	43.00 (25.00, 58.50)	52.60 ± 14.04	0.218^b^	80.00 (46.00, 85.50)	66.00 ± 19.52	0.968^b^	83.00 (78.50, 90.50)	47.80 ± 20.61	0.000^b^
Test time of test 2	241.00 (214.50,772.50)	416.10 ± 167.28	0.353^b^	334.40 ± 202.97	273.90 ± 115.00	0.423^a^	377.70 ± 284.00	345.00 ± 157.61	0.620^b^	318.00 (235.20,546.50)	269.00 (171.50, 374.75)	0.149^b^
Scores of test 2	93.00 (91.50, 96.00)	94.20 ± 4.94	0.430^b^	84.00 ± 9.66	91.60 ± 6.38	0.053^a^	92.00 (81.00,95.50)	92.90 ± 5.71	0.081^b^	93.30 ± 4.91	90.00 (79.50, 95.50)	0.038^b^
Scores improvement of test 2	8.20 ± 10.52	14.80 ± 14.67	0.263^a^	41.00 ± 23.08	39.00 ± 14.91	0.263^b^	21.40 ± 22.65	26.90 ± 19.01	0.74^a^	11.50 ± 12.88	40.00 ± 18.94	0^a^
Test time reduction of test 2	185.90 ± 442.11976	136.70 ± 312.36	0.777^a^	负48.4 ± 188.02	12.20 ± 152.39	0.777^b^	104.50 ± 351.65	74.45 ± 247.58	0.953^a^	161.30 ± 373.42	−18.10 ± 169.44	0.058^a^
Test time of test 3	94.00 (89.50, 98.50)	286.20 ± 81.46	0.684^b^	251.80 ± 96.52	214.80 ± 69.71	0.339^a^	252.50 (185.75, 315.00)	213.50 (188.00, 312.00)	0.904^b^	286.85 ± 116.80	196.00 (182.50, 264.25)	0.038^b^
Scores of test 3	93.80 ± 5.20	95.00 ± 5.27	0.615^a^	91.40 ± 5.74	91.40 ± 6.60	0.615^b^	92.30 ± 5.36	93.20 ± 6.10	0.745^a^	96.00 (90.00, 99.50)	91.40 ± 6.02	0.114^b^
Score improvement of test 3	9.60 ± 10.95	15.60 ± 13.75	0.295^a^	48.40 ± 22.31	38.80 ± 12.23	0.295^b^	25.20 ± 25.07	27.20 ± 17.38	0.800^a^	12.60 ± 12.48	43.60 ± 18.19	0^a^
Test time reduction of test 3	341.20 ± 346.48	266.60 ± 294.02	0.610^a^	34.2 ± 194.54	71.30 ± 141.96	0.610^b^	209.00 ± 312.18	103.00 (16.25, 264.00)	1.000^b^	303.90 ± 315.08	52.75 ± 166.84	0.003^a^

a Student’s *t*-test.

b Wilcoxon Mann–Whitney test.

There were no statistical differences of test time duration of test 1, scores of test 1, test time duration of test 2, scores improvement of test 2, test time reduction of test 2, test time duration of test 3, scores of test 3, score improvement of test 3, and test time reduction of test 3 between Group 1 and 2, Group 3 and 4, Group GANs OCT and real OCT(*p* > 0.05). All of these suggested the enrolled participants were of the same level and there were no differences in the teaching effect of the two educational tools.

In Group GANs OCT, scores improved from 80.0 (46.0, 85.5) to 92.0 (81.0, 95.5) 5 min after training (*p* < 0.001) and 92.30 ± 5.36 1 week after training (*p* < 0.001), while scores of tests 2 and 3 are similar (*p* = 0.055). The test time for tests 1 and 2 is similar (*p* = 0.393). Test time 3 is shorter than test 1 (*p* = 0.015), but similar to test 2 (*p* = 0.05).

**Figure 5:**
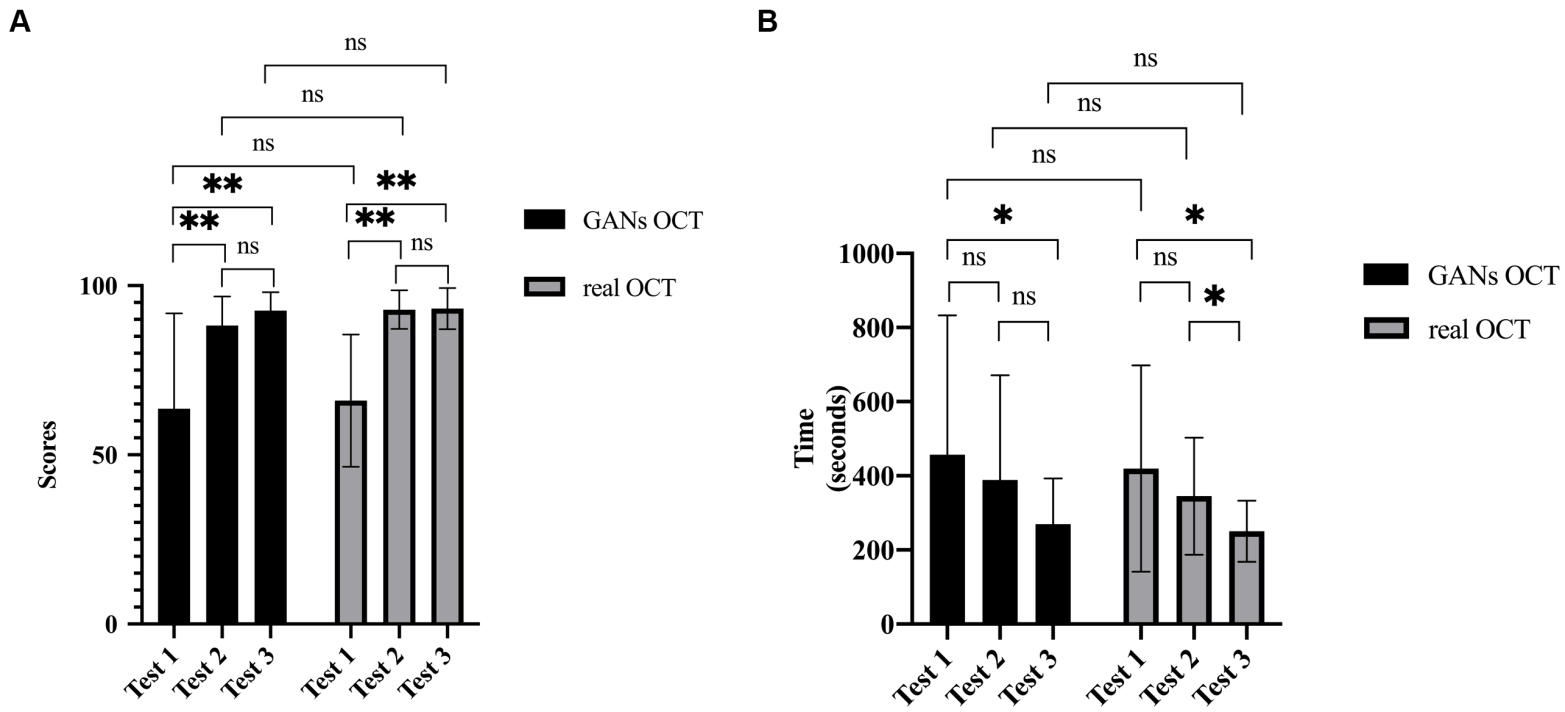
Comparisons of main results between Group GANs OCT and Group real OCT. **(A)** Scores of three tests. After the training, significant improvement was noted. No significant difference was found between Group GANs OCT and Group real OCT. **(B)** Time consumptions of three tests. After the training, with more knowledge, the participants took shorter time to finish the test. (ns=Not Statistically Significant).

In Group real OCT group, similar to that of Group GANs OCT, scores improved from 66.0 ± 19.5 to 92.9 ± 5.7(*p* < 0.001) and 93.2 ± 6.1 (*p* < 0.001), respectively, while scores of tests 2 and 3 are similar (*p* = 0.78). The test time of tests 1 and 2 is similar (*p* = 0.195). The test time of test 3 is shorter than tests 1 (*p* = 0.006) and 2 (*p* = 0.003) (Figure 5).

No significant differences were found for scores of tests, score improvements, or time consumption between Group GANs OCT and Group real OCT. Medical students had significantly higher score improvement after training than residents(*p* < 0.001).

Comparisons between Group residents and Group medical students were also performed. In terms of test scores, Group residents had higher scores on tests 1 (*p* = 0.001), 2 (*p* = 0.038), and 3, though without statistical significance (*p* = 0.114). Meanwhile, Group medical students had significantly improved scores in tests 2 (*p* < 0.001) and 3 (*p* < 0.001). In terms of test time, Group residents had longer test time in tests 1 (*p* = 0.001) and 3 (*p* = 0.038) and similar time duration in test 2 (*p* = 0.149). In terms of test time, after training, both residents and medical students took less time on test 3 than on the pretest, while medical students took more time on test 2. In terms of test time reduction, Group residents experienced more time reduction in test 3 than Group medical students (*p* = 0.003).

### Student satisfaction and evaluation

All participants responded to the questionnaires, which included two parts: part A (questions 1–8), which assessed the quality of GANs OCT images, and part B (questions 9–21), which evaluated our smartphone-based educational tool.

**Figure 6:**
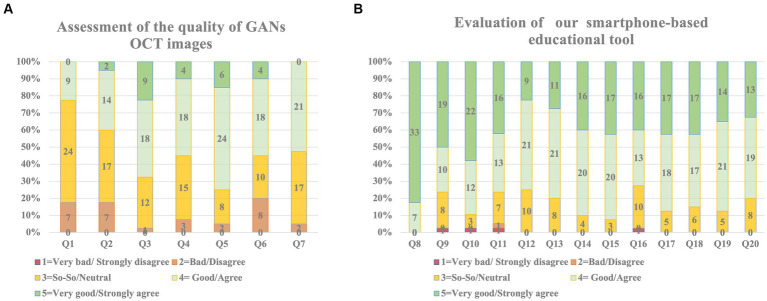
The results of the subjective questionnaires. **(A)** Results of assessment of the quality of GANs OCT images showed most of the images were of good quality. **(B)** Our smartphone-based educational tools were helpful and of high value in many aspects.

Overall, all respondents were satisfied and agreed that the GANs OCT images were of high quality and difficult to distinguish from the real OCT. They also agree that GANs OCT-based educational tool was helpful, effective, and beneficial to help improve the diagnosis ability of macular diseases (Figure 6). We also collected students’ answers to the open-ended question: What are the advantages and disadvantages of these GANs/Real OCT image-based educational tools compared to traditional teaching methods? Many students confirmed that GANs/Real OCT image-based educational tool benefits them in terms of effectiveness, convenience, plenty of impressive typical images, and good reinforcement learning of two-phase tests.

However, some participants complained about a lack of personalized study plans, interactive activities, and personalized feedback. Two participants also commented that the tool may be more suitable for beginners.

## Discussion

Resident training is crucial for every country to cultivate competitive doctors. The ACGME, a national, private, not-for-profit organization charged with accrediting medical resident training in the United States, has mandated all residency programs to implement measures to teach and assess seven core competencies: patient care, medical knowledge, professionalism, interpersonal and communication skills, practice-based learning, system-based learning and teaching, and assessing surgical skills (1). In China, the Ophthalmologist Training Committee launched standardized training programs for junior residents in 2010, much later than in developed countries. The ophthalmic resident training program was scheduled for 3 years. In phase two (years 2–3), skills to perform an OCT examination and offer a diagnosis report are essential. Traditional OCT learning included text-based teaching or hands-on training.

When all OCT images are to be used or shared between different departments or doctors, privacy rules should be considered. As defined by the US National Institute of Standards and Technology, biometric data, including retinal images, are personally identifiable information and could possibly be protected from inappropriate access regardless of the participant’s consent or local IRB permission (19). To avoid potential violations of privacy regulations, we previously proposed GANs architectures that could synthesize realistic OCT images that satisfactorily serve as training datasets for DL algorithms and education images for retinal specialists or students (20). In this study, we apply it in the real world to test its validity.

All parameters studied were similar without statistical significance between Group 1 and Group 2, Group 3 and Group 4, and Group GANs OCT and Group real OCT (*p* > 0.05). Our educational tools showed similar educational performance to real OCT image-based educational tools in terms of score improvement and test time reduction (*p* > 0.05). All subgroups experienced positive score improvements in tests 2 (short-term) and 3 (long-term), which implies the education tools’ short-term and long-term effectiveness. While in both groups, scores on tests 2 and 3 were similar, this implies these educational tools had similarly good long-term educational effects as short-term ones. Besides, the time consumption declined significantly in test 3 in both groups, which implies they were equipped with more knowledge and better competency.

To discover the tool’s educational ability among participants of different educational backgrounds, we enrolled ophthalmic residents and fifth-year medical students at the same time. Residents had more ophthalmic knowledge and got higher scores on tests 1 (*p* < 0.001) and 2 (0.038b). However, both residents and medical students had similar scores on the last test (*p* = 0.114). This illustrates that after training, all participants showed parallelly good performance. Meanwhile, medical students had an obvious score improvement in tests 2 (*p* < 0.001) and 3 (*p* < 0.001) than ophthalmic residents. This implies these educational tools may benefit more amateurs or beginners, which could be promoted more widely for educational applications in remote areas or developing countries and benefit a larger population.

We also found some interesting facts: medical students used shorter time than residents (*p* = 0.001) in tests 1 (*p* = 0.001) and 3 (*p* = 0.038), but similar time in test 2 (*p* = 0.149). In combination with the test scores, these facts may reflect the fact that medical students had no clue about the OCT diagnosis and “randomly” made the choices at first. After training, the medical student got the information and started to think and compare, which required extra time. We believe this tool has beneficial effects on motivating self-study and self-thinking.

Our anonymous questionnaire gave subjective assessments of these GANs/real OCT image-based educational tools in terms of effectiveness and convenience of knowledge acquisition, motivational dimension, and the image quality of seven GANs OCT images. Consistent with the performances of the tools, participants agreed that GANs/real OCT image-based educational tools help improve participants’ diagnostic abilities more efficiently and conveniently. The majority wanted to share the tool with others. Most participants also agreed the GANs OCT images were of good quality and hard to tell from the real ones. However, this tool needed to be further improved with a personalized study plan to make it a better one.

### Limitations

Limitations do exist in this study. Our educational tools only involved OCT images with AMD, DME, and CNV. Other macular diseases, such as macular holes, macular dystrophy, and epiretinal membranes, were not included. An updated educational tool with more comprehensive macular diseases is expected in our future study. In addition, the number of participants is relatively small, and more participants with diverse educational backgrounds should be enrolled to test the tool’s validity.

## Conclusion

In summary, our study suggested that the GANs synthetic OCT images can be used by ophthalmic resident training programs for educational applications. The education tool using synthetic OCT images had a similar educational ability compared to that using real OCT images. The GANs-based educational tool showed the advantage of promoting students’ interpretation ability of macular diseases in both short-term and long-term performances. The proposed GANs-based approaches might alleviate issues of limited medical imaging resources in universities or training institutions in remote areas or developing countries.

## Data availability statement

The original contributions presented in the study are included in the article/supplementary material, further inquiries can be directed to the corresponding authors.

## Ethics statement

The studies involving humans were approved by Institutional Review Board approval by Xinhua Hospital Affiliated to Shanghai Jiao Tong University. The studies were conducted in accordance with the local legislation and institutional requirements. The participants provided their written informed consent to participate in this study.

## Author contributions

JP: Conceptualization, Data curation, Formal analysis, Funding acquisition, Investigation, Methodology, Writing – original draft. XX: Conceptualization, Data curation, Formal analysis, Funding acquisition, Investigation, Methodology, Writing – original draft. ZL: Conceptualization, Data curation, Formal analysis, Investigation, Methodology, Writing – review & editing. YX: Conceptualization, Data curation, Formal analysis, Funding acquisition, Investigation, Methodology, Writing – review & editing. MX: Conceptualization, Data curation, Formal analysis, Writing – review & editing. LL: Conceptualization, Data curation, Writing – review & editing. HX: Conceptualization, Data curation, Resources, Writing – review & editing. HY: Data curation, Investigation, Supervision, Writing – review & editing. LC: Data curation, Investigation, Writing – review & editing. JY: Conceptualization, Software, Supervision, Visualization, Writing – review & editing. MZ: Conceptualization, Data curation, Supervision, Visualization, Funding acquisition, Writing – review & editing. PZ: Conceptualization, Funding acquisition, Investigation, Supervision, Writing – review & editing. CZ: Conceptualization, Data curation, Investigation, Supervision, Funding acquisition, Writing – review & editing.

## Footnotes

^1^https://www.ccgme-cmda.cn/news/22/1/article

^2^https://www.wjx.cn/app/exam.aspx
